# Neonatal diabetes mellitus: improved screening and early management of an underestimated disease

**DOI:** 10.1002/ccr3.1276

**Published:** 2017-11-20

**Authors:** Maëlle Wirth, Jean‐Marc Jellimann, Stéphanie Jellimann, Jean‐Michel Hascoët

**Affiliations:** ^1^ Department of Neonatology University Hospital of Nancy 54035 Nancy France; ^2^ Department of Pediatric Endocrinology University Hospital of Nancy 54500 Vandœuvre‐Les‐Nancy France

**Keywords:** Glycated hemoglobin, hyperglycemia, intrauterine growth restriction, neonatal diabetes mellitus

## Abstract

Neonatal diabetes mellitus is underdiagnosed in the neonatal period because of the metabolic adaptation capacities of the newborns. However, it is associated with increased risk of short‐ and long‐term morbidity; when transient it may recurs in adulthood. It is important to improve screening and early management with appropriate guidelines.

## Introduction

Neonatal diabetes mellitus (NDM) is considered as a rare disorder, but probably underestimated. It can be transient (TNDM) or permanent (PNDM) and is characterized by hyperglycemia within the first months of life 1, 2. Genetic defects are often associated with NDM. Associated clinical signs in TNDM are intrauterine growth restriction (IUGR) and poor weight gain. Rarely, pancreatic insufficiency may be associated with NDM 1, 2. Remission usually occurs within the first year of life, but intermittent glucose intolerance may persist. Infants may have diabetes relapse in adolescence or adulthood under conditions of increased insulin resistance 1, 2. Usually, insulin requirements are initially lower in TNDM than PNDM 1. Most cases are sporadic. The genetic disorder 6q24 is specific to TNDM which can also be caused by other genetic abnormalities 2. There is no remission with PNDM, and IUGR is less common than with TNDM 1, 2.

Mutations in KCNJ11, ABCC8, and INS genes are most commonly associated with NDM 1, 2. There are also syndromic forms of NDM associated with other mutations 1, 2, 3.

A study in mice showed that hyperglycemia increases mortality and morbidity in neonates, with particularly strong effects on brain development 4. Moreover, some mutations, as in KCNJ11 and ABCC8 genes, are often associated with neuropsychological dysfunction and development alterations 2, 5. Later on, a missed diagnosis may lead to severe unexpected consequences such as acute dehydration and acidoketosis. Given these risks, it is important to be aware of this diagnosis and diagnose it in a timely manner for appropriate management. The aim of this study was to identify clinical conditions suggestive of NDM and propose guidance in line with our previously published findings.

## Case Series

We present a case series of six patients. Data were retrospectively collected from the records of patients with NDM. In our Unit, parents are routinely informed of the possible use of medical information about their children for research purposes and provide written consent. This research has been performed in accordance with the Declaration of Helsinki.

The characteristics of the patients are presented Table 1.

**Table 1:** Characteristics of our case series

Case	Birth (GA, weight, length)	NDM/type	Age/blood glucose level at diagnosis	Clinical findings at diagnosis	Genetic diagnosis
1^a^	33 weeks GA^b^ 1270 g 36 cm	Yes/PNDM	7 h 3.84 g/L (21.12 mmol/L)	IUGR, edema, red hair, axial hypotonia, irritability, eye rolling	Negative (tested for 6q23‐25, *KCNJ11*,* ABCC8*,* INS*)
2^a^	32 weeks GA 1540 g 41 cm	Yes/TNDM (insulin stopped at 38 days of age)	20 h 2.55 g/L (14.03 mmol/L)	Red hair, no IUGR, discovery of hyperglycemia during a routine assessment of prematurity	*ABCC8* gene mutation
3^a^	35 weeks GA 1630 g /	Yes/TNDM (insulin stopped after 9 days)	2 months 4.00 g/L (22.00 mmol/L)	IUGR, discovery of hyperglycemia during gastroenteritis	*ABCC8* gene mutation
4	38 weeks GA^b^ 2110 g 45 cm	Yes/TNDM (insulin stopped at 9 months of age)	8 days 9.94 g/L (54.67 mmol/L)	Dehydration, ketoacidosis, IUGR, triangular face, large fontanel, macroglossia, protrusion of tongue, fine skin	6q24 paternal disomy
5^a^	41 weeks GA 2560 g 47 cm	Yes/TNDM (insulin stopped after 3 weeks)	3 months (recurrence at 12 years) 5.50 g/L (30.25 mmol/L)	IUGR, discovery of hyperglycemia during gastroenteritis, bronchitis, thrush	Negative for chromosome 6 and *KCNJ11*/*ABCC8* mutations, ongoing
6^a^	At term About 3000 g /	TNDM suspected a posteriori from file (weight stagnation in the first weeks of life)	30 years	PUPDS, asthenia, no IUGR	*INS* gene mutation

a Family history of diabetes.

b Cases 1 and 4 have parental consanguinity.

GA, gestational age; PUPDS, polyuria–polydipsia syndrome; IUGR, intrauterine growth restriction; NDM, neonatal diabetes mellitus; TNDM, transient neonatal diabetes mellitus; PNDM, permanent neonatal diabetes mellitus.

In our series, 3 of 6 infants were born prematurely. Four infants had TNDM, one had PNDM, and one had suspected TNDM. A diagnosis of diabetes was made before 6 months of age in five infants, and in adulthood in the last case. The diagnosis was based on diabetic symptomatology in 2 of 6 cases and was incidental in 4 of 6 (Cases 2, 3, 5, 6). Five of the six patients had a family history of diabetes, and two of the patients had consanguinity. Genetic investigations revealed that one patient had a mutation in the INS gene, two had a mutation in the ABCC8 gene, one had 6q24 disomy, and two had no identified mutations. Of the six patients, four had decreased C‐peptide and insulin blood levels, one had slightly increased levels of C‐peptide and insulin (case 3), and data were missing for the last patient.

## Discussion

We identified mutations in 4 of 6 cases (67%). It is less than in the study of Besser et al. 6, that is, probably explained by our small sample size. However, all our identified mutations are described in neonatal diabetes mellitus 6. Our findings illustrate the phenotypic polymorphism of NDM, which has a wide variability of expression with respect to the age of onset, incidental findings, IUGR, poor weight gain, and diabetic ketoacidosis. In our series, case 6 illustrates that symptoms in the neonatal period may be moderate and the diagnosis missed when clinicians are not watchful. Of note, the diagnosis was made incidentally in more than half of our cases. This can be explained by the features of newborn glucose metabolism. In term infants, carbohydrate consumption is three times higher than in adults, in particular in the brain which metabolizes 50–70% of available blood glucose for its development 7. Moreover, cerebral use of ketone bodies is 40 times higher in the newborn brain compared to adults 8. Thus, symptoms of hyperglycemia and hypoinsulinism may be minimized or remain unnoticed based on the degree of severity in the newborns. In addition, there are no pathognomonic symptoms indicative of NDM.

Neonatal diabetes mellitus diagnosis is difficult, and its incidence is underestimated. Therefore, because of the risk of recurrence associated with TNDM 1, 2, the risk of neurological morbidity associated with hyperglycemia in newborns 4, and the risk of neuropsychological dysfunction and development alterations associated with several mutations 5, it is important not to miss this diagnosis in the neonatal period. Clinicians must remain watchful for warning signs, especially family cases of diabetes, IUGR, unusually persistent high blood glucose levels, poor weight gain (sign of hypoinsulinism after birth), or dehydration (sign of ketoacidosis).

Neonatal glycemic standards are poorly defined, and plasma glucose levels are lower in neonates than in children 9, 10. In healthy term infants, blood glucose level is lower in the first hours of life and gradually increases 9, 10. Preterm infants have a greater risk of hyperglycemia 10. In preterm infants born before 32 weeks gestation (GA), neonatal diabetes may cause more severe and earlier damage than idiopathic transient hyperglycemia 11. Consistently with Hoseth et al. 9 and Hawdon et al. 10 and with the fact that none of our patients with a neonatal diagnosis had a blood glucose level lower than 11.1 mmol/L (2 g/L), we propose the following index of suspicion should be raised in the following context for NDM suspicion when family cases of diabetes, IUGR, poor weight gain, or dehydration are present as follows: a glycemic threshold in term newborns of 6.2 mmol/L (1.1 g/L) in the first week of life and then 11.1 mmol/L. Besser et al. recommend complementary investigations of preterm infants born after 32 GA with hyperglycemia 6. For preterm infants born after 32 GA, the high glycemic threshold could also be 11.1 mmol/L from birth 10. These thresholds are applicable in the absence of factors known to be associated with hyperglycemia, such as stress, infection, use of glucocorticoids or catecholamine treatment, and excessive glucose supply. As there is daily glycemic variability, a single measurement does not seem enough to suspect this diagnosis. From our point of view, it appears necessary to do at least two measurements 24 h apart before considering additional assessments: on one hand to avoid the influence of any stress event on the first result and on the other hand to prevent unnecessary blood withdrawal. For preterm infants born before 32 GA, Besser et al. suggest that complementary investigations should be realized only if hyperglycemia persists until the postmenstrual age of 32 weeks 6. Busiah et al. suggest that a blood glucose level ≥ 20 mmol/L in the first day of life or prolonged insulin requirements (they noted a median of 85 days in their study) may be indicative of NDM 11. Because of the high frequency of a family history of diabetes in patients with NDM, it could also be useful to add blood glucose screening in infants with a family history of diabetes to the routine neonatal screening.

Glycated hemoglobin (HbA1c), a marker of the glycemic status of the previous 2–3 months, cannot be used for blood glucose monitoring before the age of 6 months, because of the presence of fetal hemoglobin. Conversely, glycated albumin reflects blood glucose levels from the previous month and may be a good monitoring marker in infants with NDM who do not have any abnormalities of albumin metabolism 12. Reference values for glycated albumin levels in infants who do not have an albumin metabolism disorder were studied by Suzuki et al. and are presented in Table 2 13.

**Table 2:** Reference values for glycated albumin levels in infants who do not have an albumin metabolism disorder 13

Age	Glycated albumin (%)
0 days, from cord blood	8.3–10.5
4–7 days	4.9–9.4
7–14 days	5.5–10.1
2–4 weeks	6.2–10.8
1–2 months	6.9–11.6
3–6 months	8.0–12.7
6–12 months	8.9–13.4

**Figure 1:**
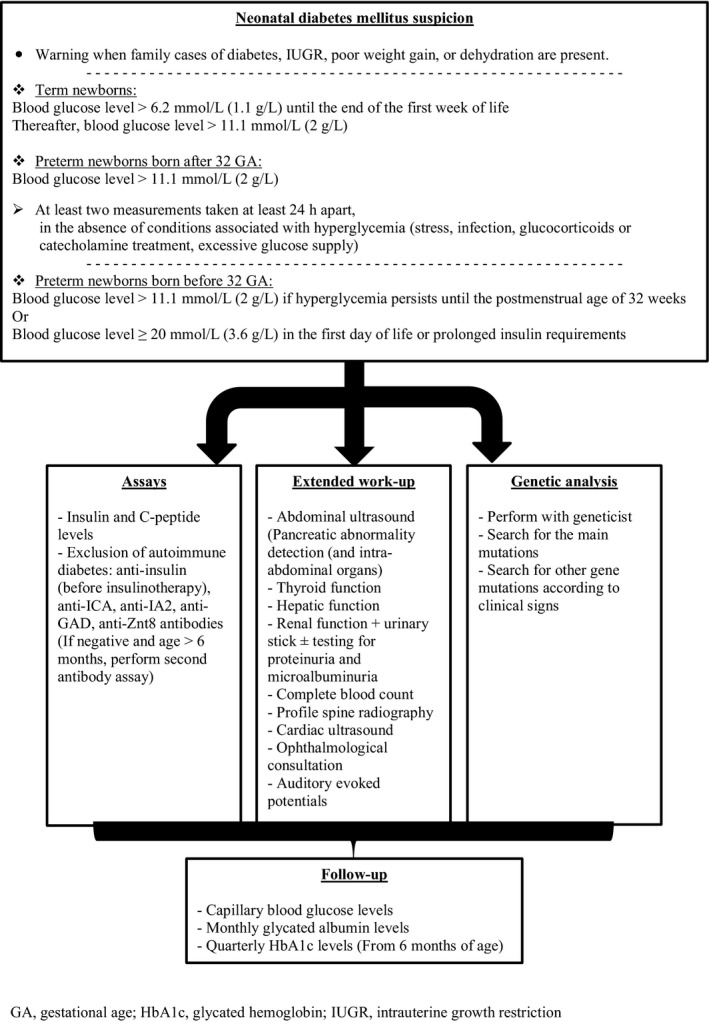
Algorithm for the detection and management of neonatal diabetes mellitus.

Once NDM is suspected, clinicians must carry out an etiology assessment and verify the absence of concomitant impairments. Anti‐ICA (islet cell antibody), anti‐IA2 (islet cell antigen), anti‐GAD (glutamate decarboxylase acid), anti‐insulin (before initiation of insulin therapy), and anti‐ZnT8 (zinc transport 8) antibodies should be evaluated and will be negative in patients with NDM. If antibodies are negative but the child is older than 6 months, a second evaluation should be performed 2. Insulin and C‐peptide levels may be measured and are needed to prove endogenous insulin deficiency, but they will not necessarily be low, as in case 3 in our series. Other examinations searching for associated disorders or syndromic forms should include 1, 2, 3 as follows: thyroid (looking for autoimmune hypothyroidism), fecal elastase (looking for associated exocrine pancreatic insufficiency), hemoglobin and platelet counts, renal and hepatic screening. Abdominal and cardiac ultrasound should be performed to evaluate pancreatic, gallbladder, and liver morphology and search for cardiac abnormalities. A spinal radiography should be performed to identify spondyloepiphyseal dysplasia. Associated eye injury and hearing loss should also be explored 1, 2, 3. Genetic analysis should be performed according with clinical signs for all children diagnosed within the first 6 months 14. Figure 1 proposes an algorithm outlining the actions to take when NDM is suspected. Clinical management should be multidisciplinary with Geneticists and Pediatric Endocrinologists involved. Treatment aims in infants with NDM are to achieve glycemic control and prevent brain damage and to achieve normal weight and height development. Adequate glucose intake should be maintained in these patients to allow good growth and brain development. Treatment comprises insulin therapy, possibly with the use of a pump 2. Sulfonylureas are more effective in patients with KCNJ11 or ABCC8 gene mutation 1, 2. Therefore, genetic analysis is mandatory in NDM, not only to confirm the diagnosis but also to guide treatment options. The major limitation of this study is the small cohort size. But, given NDM risks, it is important to propose guidance in accordance with our and also previously published findings until it is validated by a larger cohort.

## Authorship

MW: conceptualized and designed the study, carried out the acquisition of data, the initial analyses and interpretation of data, and drafted the initial manuscript. J‐MH, J‐MJ and SJ: conceptualized the study, carried out the interpretation of data, reviewed and revised the manuscript for content. All authors approved the final manuscript as submitted and agree to be accountable for all aspects of the work.

## Conflict of Interest

None declared.
